# X-ray Shielding, Mechanical, Physical, and Water Absorption Properties of Wood/PVC Composites Containing Bismuth Oxide

**DOI:** 10.3390/polym13132212

**Published:** 2021-07-04

**Authors:** Worawat Poltabtim, Ekachai Wimolmala, Teerasak Markpin, Narongrit Sombatsompop, Vichai Rosarpitak, Kiadtisak Saenboonruang

**Affiliations:** 1 Department of Applied Radiation and Isotopes, Faculty of Science, Kasetsart University, Bangkok 10900, Thailand; wp.worawat@gmail.com; 2 Polymer PROcessing and Flow (P-PROF) Research Group, Division of Materials Technology, School of Energy, Environment and Materials, King Mongkut’s University of Technology Thonburi, Bangkok 10140, Thailand; ekachai.wim@kmutt.ac.th (E.W.); teerasak.mar@kmutt.ac.th (T.M.); narongrit.som@kmutt.ac.th (N.S.); 3 V.P. Wood Co., Ltd., Suksawat 41, Banphueng, Phra Pradaeng Samut Prakan 10130, Thailand; cabonyx.sales@gmail.com; 4 Specialized Center of Rubber and Polymer Materials in Agriculture and Industry (RPM), Faculty of Science, Kasetsart University, Bangkok 10900, Thailand

**Keywords:** WPVC, Bi_2_O_3_, X-ray, shielding, radiation, mechanical properties, composites

## Abstract

The potential utilization of wood/polyvinyl chloride (WPVC) composites containing an X-ray protective filler, namely bismuth oxide (Bi_2_O_3_) particles, was investigated as novel, safe, and environmentally friendly X-ray shielding materials. The wood and Bi_2_O_3_ contents used in this work varied from 20 to 40 parts per hundred parts of PVC by weight (pph) and from 0 to 25, 50, 75, and 100 pph, respectively. The study considered X-ray shielding, mechanical, density, water absorption, and morphological properties. The results showed that the overall X-ray shielding parameters, namely the linear attenuation coefficient (µ), mass attenuation coefficient (µ_m_), and lead equivalent thickness (Pb_eq_), of the WPVC composites increased with increasing Bi_2_O_3_ contents but slightly decreased at higher wood contents (40 pph). Furthermore, comparative Pb_eq_ values between the wood/PVC composites and similar commercial X-ray shielding boards indicated that the recommended Bi_2_O_3_ contents for the 20 pph (40 ph) wood/PVC composites were 35, 85, and 40 pph (40, 100, and 45 pph) for the attenuation of 60, 100, and 150-kV X-rays, respectively. In addition, the increased Bi_2_O_3_ contents in the WPVC composites enhanced the Izod impact strength, hardness (Shore D), and density, but reduced water absorption. On the other hand, the increased wood contents increased the impact strength, hardness (Shore D), and water absorption but lowered the density of the composites. The overall results suggested that the developed WPVC composites had great potential to be used as effective X-ray shielding materials with Bi_2_O_3_ acting as a suitable X-ray protective filler.

## 1. Introduction

X-rays are ionizing radiation with energies of 100 eV–100 keV and frequencies of 10^16^–10^20^ Hz and are currently utilized in various applications, including X-ray imaging for the diagnosis of brain and lung cancers [1], low-dose X-ray radiotherapy [2], X-ray fluorescence (XRF) and X-ray diffraction (XRD) for material and archeological characterizations [3,4,5], and X-ray irradiation of economic plants to accelerate breeding and mutations [6]. Despite its great benefit and potential, excessive exposure to X-rays could harmfully affect both the radiation users and the general public, whose symptoms may vary from mild conditions (nausea, vomiting, diarrhea, fever, loss of appetite, skin burn, and hair loss) to severe conditions (cognitive impairment, seizures, electrolyte disturbance, cancers, and death) depending on the X-ray energy, exposure dose and rate, and the organ response to exposure [7,8]. Consequently, to reduce and/or prevent risks from X-ray exposure, a radiation safety principle called “As Low As Reasonably Achievable” or “ALARA” [9] must be strictly followed in all radiation-related facilities [10]; the principle involves appropriate management of working time and distance, as well as the utilization of sufficient and suitable radiation shielding equipment.

Specifically, in cases where prolonged working time and/or the need to operate close to radiation sources are unavoidable, the use of appropriate and effective radiation shielding equipment is a necessary measure to ensure safety for all users [11,12]. Generally, materials containing high contents of heavy atoms or heavy compounds such as lead (Pb) and Pb-containing compounds (PbO and Pb_2_O_3_) are commonly used as parts of X-ray shielding equipment that are both effective and economically accessible due to the relatively high atomic number (Z = 82) and density of Pb, which results in enhanced probabilities of interaction between the incident X-rays and the materials [13]. However, Pb and Pb-containing compounds are health hazardous as they could severely harm most human and animal organs as well as negatively affect plants and ecosystems [14,15,16]. To minimize the risks of Pb exposure, there has been a constant effort to identify Pb-free X-ray shielding materials having comparable or better attenuation abilities than Pb-containing materials, with additional preferred properties such as transparency, flexibility, and self-healable. Examples of recent Pb-free X-ray shielding materials are: Bi_2_O_3_/polyvinyl chloride (PVC) composites [17], W/Bi_2_O_3_/high-functional methyl vinyl silicone rubber (VMQ) composites [18], W_2_O_3_/ethylene propylene diene monomer (EPDM) composites [19], Bi_2_O_3_/wood/natural rubber (NR) composites [20], and Bi/high-density polyethylene (HDPE) composites [21], in which Bi and W compounds act as alternative X-ray protectively fillers to Pb. It should be noted that the attenuation efficiencies of the mentioned materials vary depending on the filler types, contents, and the manufacturing process of the materials [22].

Among the alternative X-ray protective fillers, Bi_2_O_3_ is promising as a replacement for Pb and Pb-containing compounds due to the high atomic number of Bi atoms (Z = 83) and the high density of Bi_2_O_3_ (ρ = 8.9 g/cm^3^), which result in high interaction probabilities between incident X-rays and shielding materials that are comparable to common Pb-containing materials [23]. For example, EPDM composites containing 500 parts per hundred parts of rubber by weight (phr) of Bi_2_O_3_ had a mean (±standard deviation) linear attenuation coefficient (µ) of 24.3 ± 2.2 m^−^^1^ at a gamma energy of 1.25 MeV [19], which was comparable with NR composites containing the same Pb content (µ = 26.0 m^−^^1^) [24]. This comparison implied a promising X-ray attenuation capability of Bi_2_O_3_ that could potentially replace Pb and Pb-containing compounds as an effective protective filler. Furthermore, Bi_2_O_3_ is considerably safer for users and the environment than Pb as evidenced by the wide application of Bi_2_O_3_ in medical and industrial applications such as being an active ingredient of Pepto-Bismal (an over-the-counter medicine for the treatment of stomach disorders) and as green Lewis acid catalysts in organic synthesis, respectively [25].

In addition to X-ray protective fillers, selection of the main matrix also plays an important role in defining the useability of the developed materials in actual applications, because other important properties such as mechanical strength, water absorption, and flexibility, of the composites largely depend on the properties of the main matrix [26,27]. In particular for X-ray shielding materials used as movable panels, radio-diagnostic walls, and transportation casks, wood/plastic composites (WPC), especially wood/PVC (WPVC) composites, are most interesting for such purposes due to their light weight, low water absorption, termite resistance, humidity resistance, environmental erosion resistance, dimensional stability, and low maintenance requirements, resulting in their broad potential for utilization [28,29,30,31]. Despite their attractive properties, the use of WPC as radiation shielding material, especially for X-ray attenuation, is presently limited as most of available products and reports have been primarily aimed at preventing the interference of low-energy electromagnetic (EM) waves (radio and microwaves) on electrical devices. Examples of WPC used as electromagnetic interference (EMI) shielding materials are graphene/WPC and Ni/wood/PVC composites, which were used as EMI shields for EM waves with frequencies of 60 MHz–1.5 GHz and 8.2 GHz–12.4 GHz, respectively [32,33]. 

As aforementioned, the current work aimed to expand the limited information/data on WPC, especially on WPVC composites, in X-ray shielding applications by determining the X-ray attenuation, mechanical, morphological, density, and water absorption properties of WPVC composites containing Bi_2_O_3_ particles. The contents of wood particles were varied from 20 to 40 parts per hundred parts (pph) of PVC by weight and the Bi_2_O_3_ contents were varied from 0 to, 25, 50, 75, and 100 pph. The X-rays used for the measurement of shielding properties were generated from an X-ray tube with supplied voltages of 60, 100, and 150 kV, and the following X-ray shielding properties were determined and reported: µ, the mass attenuation coefficient (µ_m_), and the Pb equivalent thickness (Pb_eq_). In addition, recommended Bi_2_O_3_ contents for each X-ray energy were determined. The outcomes of this work should not only present novel, effective, and safe X-ray shielding materials from Bi_2_O_3_/WPVC composites but also broaden the data availability of WPVC-based materials for future development as improved radiation shielding materials.

## 2. Experimental

### 2.1. Materials and Chemicals

**Figure 1:**
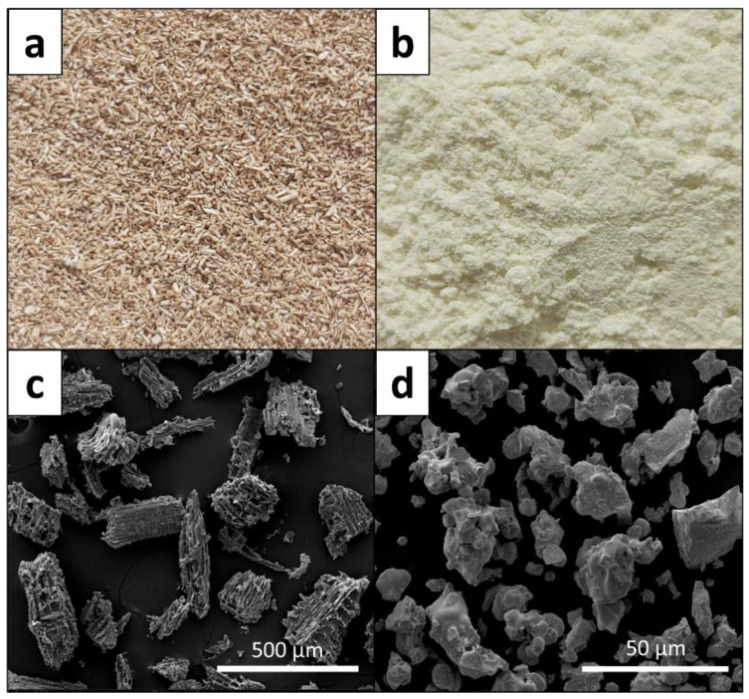
Optical images of (**a**) wood particles and (**b**) Bi_2_O_3_ particles, and micrograph images of (**c**) wood particles and (**d**) Bi_2_O_3_ particles.

Suspension PVC powder (trade name SIAMVIC-258RB) with a K value of 58 was used as the main matrix for this work. Other chemicals, with their functions, contents, and suppliers, for the production of WPVC composites are given in Table 1. Optical and micrograph images of wood particles and Bi_2_O_3_ taken using a scanning electron microscope are shown in Figure 1. It should be noted that their mean (±standard deviation) particle sizes were 0.5 ± 0.1 mm and 27.4 ± 8.2 µm, respectively, determined using their respective micrograph images in the Image J software (version 1.50i).

### 2.2. Preparation of WPVC Composites

The wood particles were chemically surface-treated with a silane coupling agent (KBM603) solution using a high-speed mixer at 1000 rpm for 5 min and dried in a hot-air oven (GT-7017-L, Gotech Testing Machine, Taichung City, Taiwan) at 80 °C for 72 h until constant weight was achieved. It should be noted that the treatment of the wood particles followed procedures optimized by our previous works [34]. Then, the surface-treated wood particles were mixed with the suspension PVC and other chemicals (Table 1) using a high-speed mixer at 1000 rpm for 5 min. The mixtures were then melt-blended using a twin-screw extruder (CTW 100 QC, HAAKE™ Rheomex, Kahlsruhe, Germany) with a screw speed of 40 rpm and temperature settings of 140, 150, 160, and 160 °C for the feed zone, plastification zone, mixing zone, and die zone, respectively. The extrudate was then pelletized into granules and dried in a hot-air oven at 80 °C overnight to completely remove moisture. The dried granules were then molded into specimens using hot compression molding (LP-20M; Labtech Engineering Co., Ltd., Bangkok, Thailand) at 170 °C and pressure of 150 kg/cm^2^ for 8 min. It should be noted that the molds used in this work were 15 cm × 15 cm with two different thicknesses (3 mm and 10 mm).

### 2.3. Characterization

#### 2.3.1. X-ray Shielding Measurement

**Figure 2:**
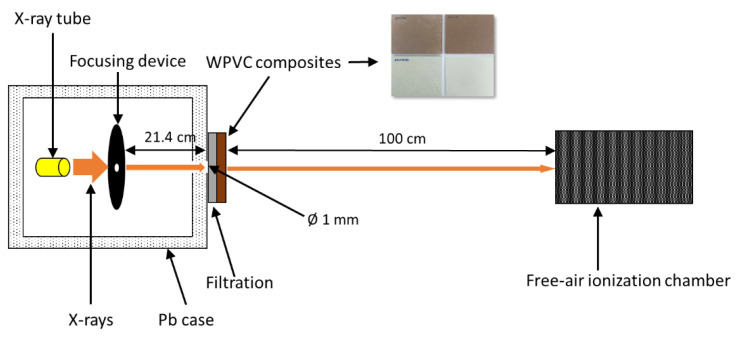
Experimental setup for X-ray shielding measurement.

The X-ray shielding properties of the WPVC composites were characterized by determining the values of the X-ray transmission ratio (I/I_0_), the linear attenuation coefficient (μ), the mass attenuation coefficient (μ_m_), and the lead (Pb) equivalent thickness (Pb_eq_) [12]. The tests were performed at the Secondary Standard Dosimetry Laboratory (SSDL), the Office of Atoms for Peace (OAP), Bangkok, Thailand, using the experimental setup shown in Figure 2.

To perform the X-ray shielding measurement, X-rays, generated using an X-ray tube with supplied voltages of 60, 100, and 150 kV, and collimated using a 1 mm Pb pinhole, were directed at the center of 10 mm-thick WPVC samples. The transmitted X-rays were then detected and counted using a free air ionization chamber (Korea Research Institute of Standards and Science, KRISS; Daejeon, Korea), which was installed on the calibration bench in the setup. It should be noted that the detector was powered by a high voltage power supply (Keithley 247, Cleveland, OH, USA) and connected to an electrometer (Keithley 6517B, Cleveland, OH, USA) to complete the detection system. The X-ray source used in this work was controlled by X-ray systems (YXLON MGC41, Hudson, NY, USA) and their energies were selected based on the standard method recommended by ISO4037-1:2019.

To determine I/I_0_, µ, µ_m_, and Pb_eq_, three independent 5 min tests were performed and their values were calculated using Equations (1)–(3):

(1) $$I=I_{0}e^{-\mu x}$$

(2) $$\mu_{m}=\mu \rho \;$$

(3) $$\mu_{Pb}\times Pb_{eq}=\mu_{WPVC}\times x$$

where I, I_0_, x, ρ, µ_Pb_, and µ_WPVC_ are the intensity of transmitted X-rays, the intensity of the incident X-rays, the thickness of the WPVC samples, the density of the WPVC samples, the linear attenuation coefficient of Pb, and the linear attenuation coefficient of the WPVC samples, respectively. For comparative purposes, a pure Pb sheet was also tested using the same testing procedure and setup, and its I/I_0_ and µ_Pb_ were calculated and reported.

#### 2.3.2. Mechanical Properties

The flexural strength of the WPVC composites was investigated using a universal testing machine (The Starrett FMS5000; Lynchburg, VA, USA), following the ASTM D790-10 standard testing. The Izod impact strength of the WPVC composites was measured according to ASTM D256-10, using a pendulum impact testing machine (United Test JB-300B; Beijing United Test Co., Ltd., Beijing, China). For hardness (Shore D) measurement, all samples were tested according to ASTM D2240-05 standard testing using a hardness durometer (Shore D) (Teclock GS-720N, Nagano, Japan). It should be noted that all mechanical measurements were conducted with at least three specimens for each formulation.

#### 2.3.3. Morphology and Density Measurement

The morphology and dispersion of the Bi_2_O_3_ and wood particles in the WPVC composites were determined using scanning electron microscopy (SEM) and energy dispersive X-ray spectroscopy (EDX) (Quanta 450 FEI: JSM-6610LV, Eindhoven, The Netherlands) at a 10-kV accelerating voltage. Prior to the SEM-EDX images being taken, all specimens were coated with gold using a sputter coater (Quorum SC7620: Mini Sputter Coater/Glow Discharge System, Laughton, UK) at a power voltage of 10 kV and a current of 10 mA for 120 s. 

The density (ρ) of each sample was determined by finding the ratio of the mass (m) to the volume (V) of the specimen [11] and the results are shown in Table 2, which indicates that the densities tended to increase with increasing Bi_2_O_3_ contents but slightly decreased with increasing wood contents. This could have been due to the much higher density of Bi_2_O_3_ (ρ_Bi2O3_ = 8.9 g/cm^3^) [12,19,23] compared to PVC (ρ_PVC_ = 1.43 g/cm^3^) [35] that resulted in the higher overall densities of the WPVC composites after the addition of Bi_2_O_3_ into the composites. In contrast to Bi_2_O_3_, the addition of wood particles slightly reduced the overall densities of the composites as the wood particles had much lower densities than Bi_2_O_3_ and PVC (ρ_wood_ = 0.48–0.65 g/cm^3^) [36], resulting in an approximately 2% decrease in the densities of the WPVC composites containing 40 pph wood particles compared to those with 20 pph wood particles.

#### 2.3.4. Water Absorption Measurement

The measurement of water absorption for all WPVC composites was performed following ASTM D570-98 (2018) standard testing, with at least three specimens for each formulation tested. The WPVC specimens were dried in a hot-air oven (GT-7017-L, Gotech Testing Machine, Taiwan) at 50 °C for 24 h to achieve a constant weight and then immersed in a deionized water bath for 24 h. The specimens were taken out of the water, wiped with tissue paper to remove surface water, and immediately reweighed on a balance with precision of 0.0001 g. The percentage of water absorption was then calculated by finding the ratio of the weight difference between the sample submerged in water (W_s_) and the dried sample (W_d_) to the weight of the dried sample (W_d_) as shown in Equation (4):

(4) $$Water\;absorption\;\left(\%\right)=\frac{W_{s}-W_{d}}{W_{d}}\times 100$$

## 3. Results and Discussion

### 3.1. Mechanical Properties

**Figure 3:**
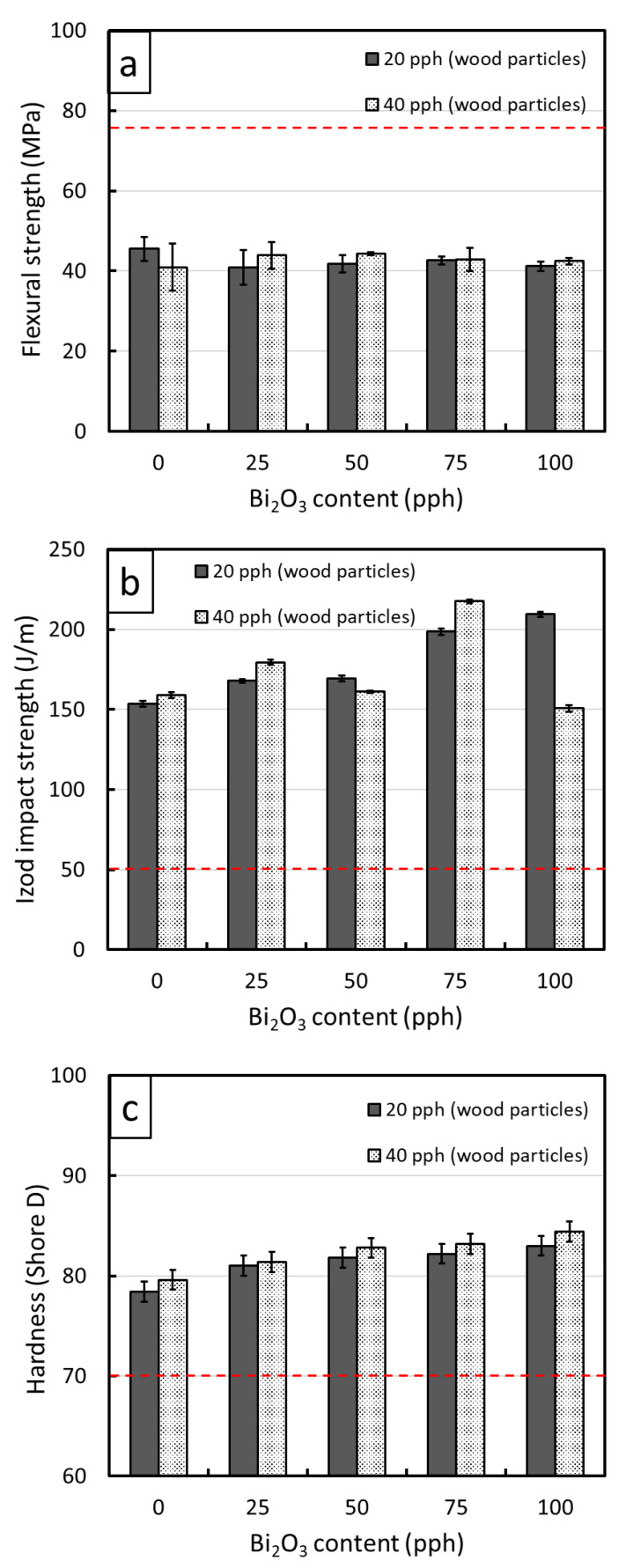
(**a**) Flexural strength, (**b**) Izod impact strength, and (**c**) hardness (Shore D) of WPVC composites containing 0–100 pph of Bi_2_O_3_ and 20–40 pph of wood particles, where error bars = ± standard deviation. The red-dashed lines represent flexural strength, Izod impact strength, and hardness (Shore D) of a neat PVC obtained from [37,38,39].

Mechanical properties, namely the flexural strength, the Izod impact strength, and the hardness (Shore D), of the WPVC composites containing varying contents of Bi_2_O_3_ are shown in Figure 3. The results indicated that values of flexural strength for all samples were not statistically different (the values fluctuated within their standard deviations), with the values being in the range 40.92–45.49 MPa. This behavior was observed due to the wood particles, for which their surfaces were chemically treated using a silane coupling agent, exhibiting improved compatibility with the PVC matrix, as well as the added Bi_2_O_3_ being uniformly dispersed throughout the matrix by the high shear stress of a twin-screw extruder. In addition, the results suggested that the values of the Izod impact strength and hardness (Shore D) were enhanced with the addition of Bi_2_O_3_, except for the WPVC composites containing 100 pph Bi_2_O_3_ and 40 pph wood particles that had noticeably lower Izod impact strength than those with 75 pph Bi_2_O_3_ and 40 pph wood particles (approximately 30% decrease). Similar to Bi_2_O_3_, the samples having a higher wood content (40 pph) generally had higher overall mechanical strength at the same Bi_2_O_3_ content than those with 20 pph wood particles, implying the role of wood particles in the WPVC composites as a reinforcing filler. 

**Figure 4:**
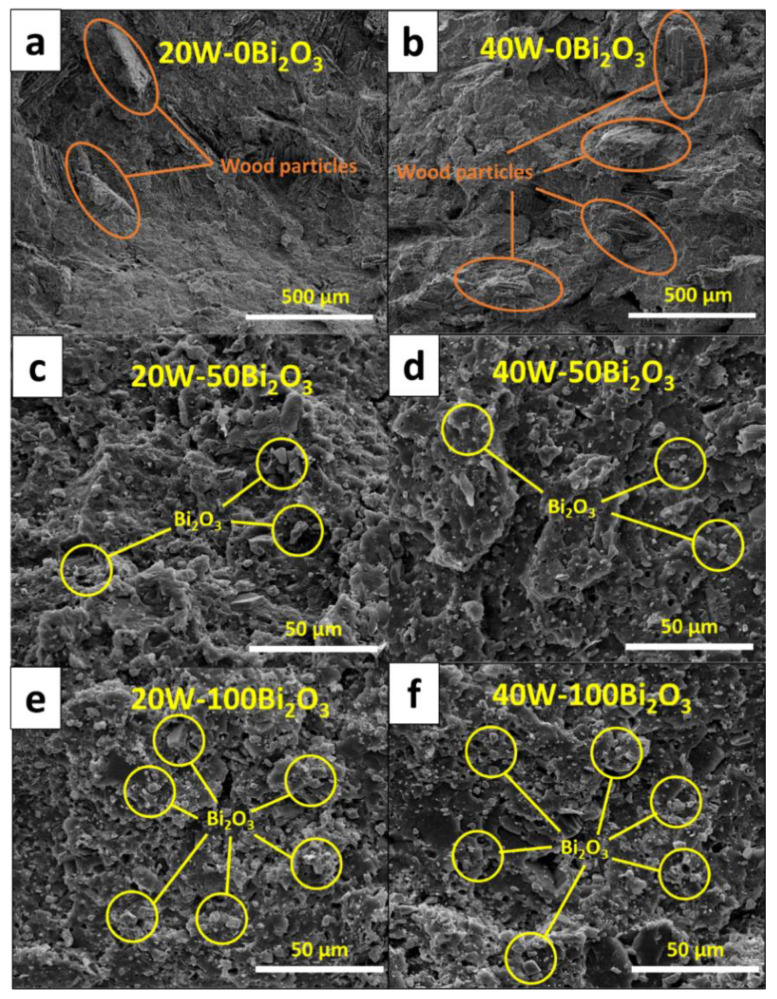
SEM images of WPVC composites containing (**a**) 20 pph wood particles, (**b**) 40 pph wood particles, (**c**) 50 pph Bi_2_O_3_ and 20 pph wood particles, (**d**) 50 pph Bi_2_O_3_ and 40 pph wood particles, (**e**) 100 pph Bi_2_O_3_ and 20 pph wood particles, and (**f**) 100 pph Bi_2_O_3_ and 40 pph wood particles.

The increases in the Izod impact strength with the addition of Bi_2_O_3_ and wood particles, which was as high as 217.5 ± 1.3 J/m in the sample with 75 pph Bi_2_O_3_ and 40 pph wood particles, could have been due to both fillers in the matrix limiting chain segmental motions and consequently reducing the flexibility of the matrix reins that required higher energy to fracture the WPVC composites [40,41]. However, for the composites with very high filler contents, such as with 100 pph Bi_2_O_3_ and 40 pph wood particles, the Izod impact strength was considerably lower than those having less filler due to increases in the agglomeration of fillers in the PVC matrix that resulted in increased numbers of defects and voids. To illustrate the effects of a high filler content on the morphology of a composite, SEM images showing filler distribution as well as particle agglomeration in different composites are shown in Figure 4, which indicates that the composites with 100 pph Bi_2_O_3_ (Figure 4e,f) clearly had more voids in the matrix than the neat WPVC composites (Figure 4a,b) and those containing 50 pph of Bi_2_O_3_ (Figure 4c,d), resulting in a substantially reduced Izod impact strength. It was notable that the observed behaviors were in agreement with previous reports of SiO_2_/epoxy and graphene oxide/epoxy composites for which the impact strength decreased at high filler contents caused by the agglomeration of the fillers [40,41]. In addition to the flexural and Izod impact strengths, Figure 3c suggested that the hardness (Shore D) of the WPVC composites increased with increasing Bi_2_O_3_ and wood contents. This behavior was observed due to the high rigidity of the Bi_2_O_3_ and wood particles that enhanced the overall rigidity and, subsequently, surface hardness of the WPVC composites [19,42].

Figure 3 also shows mechanical properties of a neat PVC (dashed lines), indicating that the values of flexural strength of WPVC composites were lower than the neat PVC, which could be due to incompatibility between the PVC matrix and the wood particles as well as differences in shrinkage of the matrix and wood particles during cooling, which generated defects and voids within the composites [37]. On the other hand, the Izod impact strength and hardness (Shore D) of the WPVC composites were noticeably higher than the neat PVC, mainly due to the reduced chain segmental motions caused by the added fillers and the high rigidity of the fillers, respectively.

### 3.2. Water Absorption

**Figure 5:**
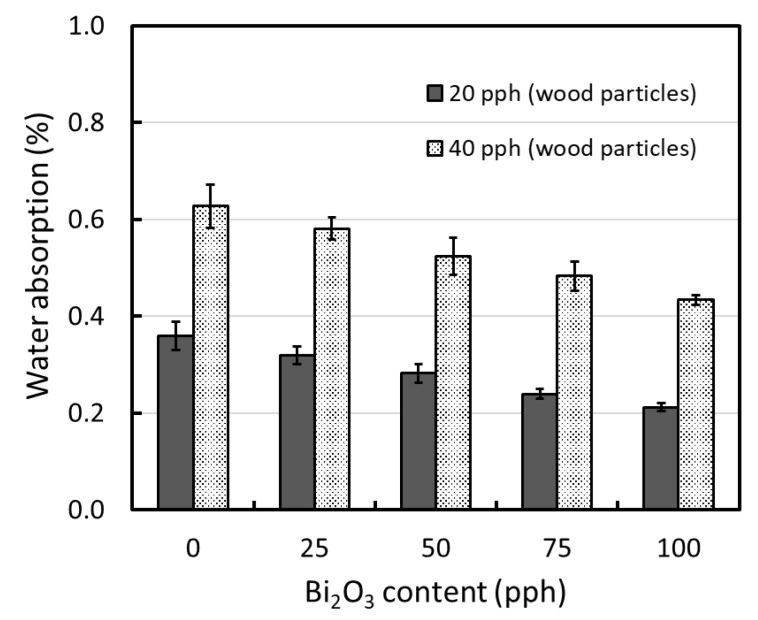
Water absorption of WPVC composites containing 0–100 pph of Bi_2_O_3_ and 20–40 pph of wood particles, where error bars = ± standard deviation.

The results for the determination of water absorption in the WPVC composites containing varying contents of Bi_2_O_3_ and wood particles are shown in Figure 5, which revealed that the percentage of water absorption decreased (increased) with increasing Bi_2_O_3_ (wood) contents. For example, samples containing 40 pph wood particles had approximately two times higher water absorption than those containing 20 pph wood particles at the same Bi_2_O_3_ content, due to the increased numbers of hydroxyl groups in the wood fiber structure that enhanced the overall hydrophilicity and, subsequently, the water absorption of the former composites [31]. In contrast, increases in the Bi_2_O_3_ contents resulted in lower water absorption of the WPVC composites. This could have been due to the dilution effects of Bi_2_O_3_, which had lower hydrophilicity than the wood particles as seen by greater water contact angle of ~107° in Bi_2_O_3_ [43] than those of ~65° in wood particles [44], which resulted in a lower weight fraction of wood particles in the composites and, consequently, reduced the overall hydrophilicity and water absorption of the composites [42].

### 3.3. X-ray Shielding Properties

**Figure 6:**
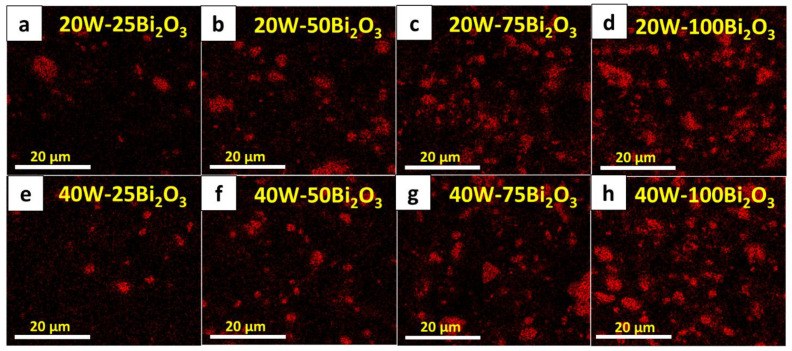
SEM-EDX images showing dispersion of Bi element in WPVC composites with (**a**) 20 pph wood particles and 25 pph Bi_2_O_3_, (**b**) 20 pph wood particles and 50 pph Bi_2_O_3_, (**c**) 20 pph wood particles and 75 pph Bi_2_O_3_, (**d**) 20 pph wood particles and 100 pph Bi_2_O_3_, (**e**) 40 pph wood particles and 25 pph Bi_2_O_3_, (**f**) 40 pph wood particles and 50 pph Bi_2_O_3_, (**g**) 40 pph wood particles and 75 pph Bi_2_O_3_, and (**h**) 40 pph wood particles and 100 pph Bi_2_O_3_. Red areas represent presence of Bi in composites.

Table 3 shows the values of µ and µ_m_ (representing the fraction of attenuated incident X-rays in a monoenergetic beam per unit thickness and unit mass, respectively) in the WPVC composites containing 0–100 pph of Bi_2_O_3_ and 20–40 pph of wood particles. The results indicated that increases in the Bi_2_O_3_ content led to the overall enhancement of X-ray shielding properties as seen by the notable increases in the values of µ and µ_m_ at higher Bi_2_O_3_ contents. The most pronounced increases in µ and µ_m_ were observed when Bi_2_O_3_ was initially added to neat WPVC composites (from 0 to 25 pph), resulting in approximately a two-times improvement in the µ and µ_m_ values. The positive dependency of the X-ray shielding abilities on the Bi_2_O_3_ contents was mainly due to the effective role of Bi_2_O_3_ in the enhancement of X-ray attenuation, with Bi_2_O_3_ acting as an X-ray attenuator that increased the interaction probabilities between the incident X-rays and the materials [22,24]. To show the distribution of Bi elements in the WPVC composites, especially for those having high Bi_2_O_3_ contents, SEM-EDX images of all samples containing Bi_2_O_3_ are shown in Figure 6, which revealed that there were clearly more Bi atoms (shown as red areas) in the samples having higher Bi_2_O_3_ contents, especially in Figure 6g,h. Thus, there was a higher X-ray shielding capability because there were more available Bi atoms to interact with X-rays.

On the other hand, Table 3 showed that increasing the wood content from 20 pph to 40 pph resulted in slightly lower values of µ and µ_m_, although the reduced values were less than 10% (determined for the same Bi_2_O_3_ content). This lower X-ray shielding property for samples with more wood particles added to the composite was due to the much lower X-ray interaction probability for the wood particles (mostly comprised of C, H, and O) compared to Bi_2_O_3_ (µ_m-wood_ = 0.162 cm^2^/g and µ_m-Bi2O3_ = 5.162 cm^2^/g for 100 keV X-rays [45]), resulting in suppressed effects of Bi_2_O_3_ in X-ray attenuation and thus, less X-ray shielding capability for those samples containing 40 pph wood particles [20]. Additionally, notable from Table 3 was that the values of µ and µ_m_ decreased at higher X-ray energies (determined at the same Bi_2_O_3_ and wood contents). This could have been due to higher-energy X-rays being less likely to interact with materials through dominant and effective photoelectric absorption, which rapidly decreased with increasing X-ray energies/frequencies, as depicted in Equation (5) [20,46]:

(5) $$\sigma_{pe}\propto \;\frac{Z^{n}}{{\left(h\nu \right)}^{3}}$$

where σ_pe_ is the photoelectric cross section, Z is the atomic number of the element, h is Planck’s constant, and ν is the frequency of the X-rays that is directly related to X-ray energy through Equation (6):

(6) E=hν

It is also notable that the values of µ and µ_m_ for a neat PVC, calculated using a web-based software (XCOM) [20,47], were similar to WPVC composites without the addition of Bi_2_O_3_, due to the low σ_pe_ of C, H, and Cl in PVC, leading to low interaction probabilities between the X-rays and the neat PVC.

**Figure 7:**
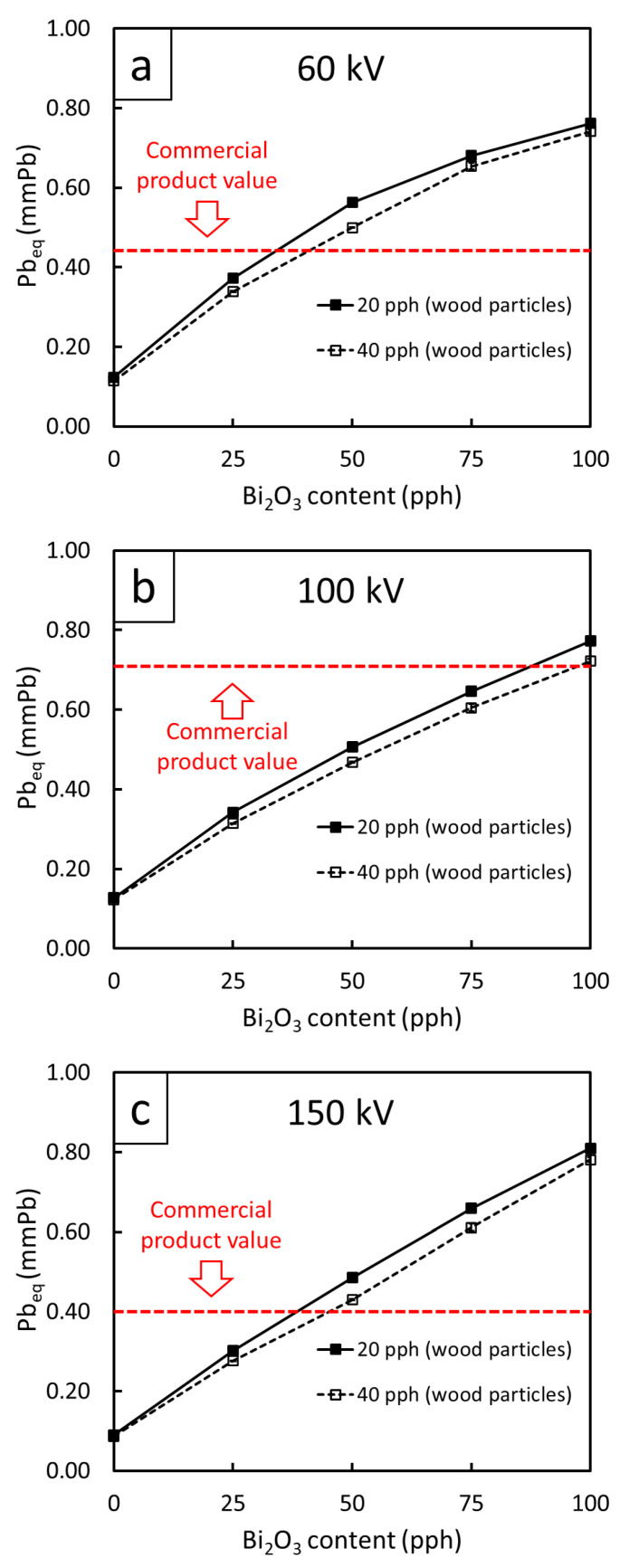
Lead equivalent thickness (Pb_eq_) of WPVC composites containing 0–100 pph of Bi_2_O_3_ and 20–40 pph of wood particles for X-rays generated from a tube with supplied voltages of (**a**) 60 kV, (**b**) 100 kV, and (**c**) 150 kV. Red dotted lines represent Pb_eq_ values of similar commercial X-ray shielding products used for determination of recommended Bi_2_O_3_ contents.

The Pb equivalent thickness (Pb_eq_), which represents the thickness of material of concern affording the same X-ray attenuation as a Pb sheet with a certain thickness (under the same specified conditions such as X-ray energies and beam sizes) at 60, 100, and 150-kV X-rays for all WPVC composites are shown in Figure 7. The results revealed that the dependence of Pb_eq_ on Bi_2_O_3_ was similar to those of µ and µ_m_ (Table 3) as the Pb_eq_ values increased with increasing Bi_2_O_3_ content but decreased with increasing wood content/X-ray energy. For example, the Pb_eq_ values of the 10 mm-thick samples were as high as 0.8 mmPb in those containing 100 pph Bi_2_O_3_, which were considerably greater than for the neat WPVC composites (0 pph Bi_2_O_3_) that only had approximately 0.1 mm Pb.

In order to determine recommended Bi_2_O_3_ contents in the WPVC composites for actual use, especially in medical applications, Pb_eq_ values obtained from the datasheets of three commercial X-ray shielding products based on a gypsum board (XRoc board) [48], plasterboard (GIB X-Block board) [49] and Knauf Safe board [50], for which the average Pb_eq_ values of the commercial products were 0.45, 0.70, and 0.40 mm Pb for the 60, 100, and 150-kV X-rays, respectively, were compared with those from the current work. It should be noted that the Pb_eq_ values of the commercial products for 100 kV X-rays were higher than other X-ray energies due to the sharp increase in X-ray interaction probabilities at the K-edge absorption of barium (Ba), which was used as the main X-ray protective filler for the commercial products, that occurs at 37.4 keV [20]. Hence, the 100 kV X-rays, with their average energy around 40–60 keV depending on the type and setup of the X-ray machine, were just above the binding energy of the electron K shells inside the Ba atoms, leading to immensely enhanced probabilities of X-ray interaction through photoelectric absorption and, subsequently, higher Pb_eq_ values at these particular energies [51]. 

As shown in Figure 7, the Pb_eq_ values from the commercial products (represented as red dotted lines) intercepted with the lines of Pb_eq_ values from the current work at different Bi_2_O_3_ contents depending on the X-ray energy and wood content. These interception points between the two lines implied the least Bi_2_O_3_ content that could produce the same X-ray attenuation ability as from commercial products, which could be regarded as the recommended Bi_2_O_3_ contents for actual production and use. The results from the determination of the recommended Bi_2_O_3_ contents for all X-ray energies are shown in Table 4, which indicated that the 60 kV and 150 kV X-rays required similar Bi_2_O_3_ contents of 35–40 pph and 40–45 pph, while the 100 kV X-rays required the highest Bi_2_O_3_ contents of 85 pph and 100 pph, for the WPVC composites containing 20 pph and 40 pph wood particles, respectively. Notably, the recommended Bi_2_O_3_ contents for the 100 kV X-rays were higher than for the other X-ray energies due to the higher Pb_eq_ values of the commercial products used as references. Notably, the recommended Bi_2_O_3_ contents for WPVC composites containing 40 pph wood particles were higher than those containing 20 pph wood particles due to the suppressed effects of Bi_2_O_3_ particles in attenuating the incident X-rays due to the greater number of wood particles in the composites. 

Lastly, the comparative X-ray shielding properties of the WPVC composites and previously reported composites containing Bi_2_O_3_ with similar filler contents and X-ray energies are shown in Table 5. The results indicated that the WPVC composites in the current work could attenuate X-rays with higher efficiencies than silicone rubber (SR) containing 50 wt.% of Bi_2_O_3_ but with slightly lower efficiencies than 50 wt.%-Bi_2_O_3_/natural rubber latex (NRL) and 35 wt/%-Bi_2_O_3_/epoxy composites. The lower µ_m_ values in the WPVC composites could have been due to the WPVC composites being investigated at higher X-ray energies as well as having less Bi atoms dispersed inside the matrix than those in the NRL and epoxy composites, which was possibly caused by fewer filler contents, the type of the main matrix, and the processing method. Nonetheless, despite their lower X-ray shielding properties, the WPVC composites were more rigid and stronger than the NRL and SR composites, making the former suitable in movable partition walls and transportation casks. Furthermore, the WPVC composites also promoted the use of natural products, which can help in reducing agricultural and industrial wastes.

## 4. Conclusions

This work developed X-ray shielding materials from WPVC composites containing Bi_2_O_3_, with varying contents of wood particles from 20 to 40 pph and of Bi_2_O_3_ from 0 to 100 pph in 25 pph increments. The results suggested that an increased Bi_2_O_3_ content led to non-statistically differences in flexural strength (the values fluctuated in the range 40.92–45.49 MPa), increased X-ray attenuation, Izod impact strength, hardness (Shore D), and density, but decreased water absorption. Furthermore, the results showed that an increased wood content tended to increase the Izod impact strength, hardness (Shore D), and water absorption, but to slightly decrease the X-ray attenuation and density of the WPVC composites. A comparison of the Pb_eq_ values obtained from the current work with similar commercial X-ray shielding products for 60, 100, and 150-kV X-rays indicated that the 60 kV and 150 kV X-rays required Bi_2_O_3_ contents of 35–45 pph, while the 100 kV X-rays required Bi_2_O_3_ contents of 85–100 pph for these WPVC composites to attenuate X-rays with the same levels of efficiency as the referenced products. Lastly, a comparison of X-ray shielding properties between the WPVC composites in the current work and other composites containing Bi_2_O_3_ with similar filler contents and X-ray energies revealed that the former could offer comparable or better X-ray attenuation than the latter for the same range of X-ray energy, indicating that the current work was successful in developing WPVC composites that had both sufficient X-ray shielding and high strength for actual production and use.

## Figures and Tables

**Table 1:** Material formulations of WPVC composites and chemical names, contents, functions, and suppliers.

Chemicals	Content (pph)	Function	Supplier
Suspension PVC grade SIAMVIC-258RB	100	Matrix	Vinythai Public Co., Ltd. (Rayong, Thailand)
Emulsion PVC grade SIAMVIC-167GZ	4.0	PVC additive	V.P. Wood Co., Ltd. (Samut Prakan, Thailand)
PF 608A, Pb-Ba based organic	3.6	Thermal stabilizer	V.P. Wood Co., Ltd. (Samut Prakan, Thailand)
PF 601	1.5	Thermal stabilizer	V.P. Wood Co., Ltd. (Samut Prakan, Thailand)
Finalux G-741	0.6	External lubricant	V.P. Wood Co., Ltd. (Samut Prakan, Thailand)
Calcium stearate	0.6	Internal lubricant	V.P. Wood Co., Ltd. (Samut Prakan, Thailand)
Calcium carbonate, Omyacarb-2T	12.0	Filler	V.P. Wood Co., Ltd. (Samut Prakan, Thailand)
Modified Chlorinated Polyethylene (CPE)	7.7	Impact modifier	V.P. Wood Co., Ltd. (Samut Prakan, Thailand)
PA-20	6.0	Processing aid	V.P. Wood Co., Ltd. (Samut Prakan, Thailand)
Para rubber wood particles	20 and 40	Filler	V.P. Wood Co., Ltd. (Samut Prakan, Thailand)
N-2-(Aminoethyl)-3-aminopropyltrimethoxysilane (KBM603)	1 wt.% of wood	Silane coupling agent	Shin-Etsu Chemical Co., Ltd., (Tokyo, Japan)
Bismuth oxide; Bi_2_O_3_	0, 25, 50, 75, and 100	X-ray protective agent	Shanghai Ruizheng Chemical Technology Co., Ltd. (Shanghai, China)

**Table 2:** Densities of WPVC composites containing varying contents of Bi_2_O_3_ and wood particles (0–100 pph in 25 pph increments and 20 to 40 pph, respectively), where results are shown as mean ± standard deviation of the mean.

Bi_2_O_3_ Content (pph)	Density (g/cm^3^)	
	20 pph Wood	40 pph Wood
0	1.46 ± 0.01	1.43 ± 0.02
25	1.63 ± 0.02	1.60 ± 0.02
50	1.79 ± 0.03	1.78 ± 0.01
75	1.97 ± 0.01	1.90 ± 0.03
100	2.09 ± 0.04	2.04 ± 0.01

**Table 3:** X-ray shielding properties, including linear attenuation coefficients (µ) and mass attenuation coefficient (µ_m_) of WPVC composites containing 0–100 pph of Bi_2_O_3_ and 20–40 pph of wood particles, Pb sheet, and a neat PVC, determined for X-ray levels of 60, 100, and 150-kV. Results are shown as mean ± standard deviation.

Wood Content (pph)	Bi_2_O_3_ Content (pph)	µ (cm^−1^)			µ_m_ (cm^2^/g)		
		60 kV	100 kV	150 kV	60 kV	100 kV	150 kV
0	0	1.02	0.36	0.25	0.73	0.26	0.18
20	0	0.84 ± 0.01	0.35 ± 0.01	0.29 ± 0.01	0.57 ± 0.01	0.24 ± 0.01	0.20 ± 0.01
	25	2.48 ± 0.01	0.94 ± 0.01	0.95 ± 0.01	1.53 ± 0.01	0.58 ± 0.01	0.58 ± 0.01
	50	3.74 ± 0.01	1.39 ± 0.01	1.51 ± 0.01	2.08 ± 0.01	0.77 ± 0.01	0.84 ± 0.01
	75	4.48 ± 0.04	1.75 ± 0.03	2.03 ± 0.01	2.27 ± 0.02	0.89 ± 0.02	1.03 ± 0.01
	100	5.04 ± 0.07	2.11 ± 0.01	2.51 ± 0.01	2.41 ± 0.03	1.01 ± 0.01	1.20 ± 0.01
40	0	0.76 ± 0.01	0.34 ± 0.01	0.27 ± 0.01	0.53 ± 0.01	0.23 ± 0.01	0.19 ± 0.01
	25	2.23 ± 0.01	0.85 ± 0.01	0.85 ± 0.01	1.40 ± 0.01	0.53 ± 0.01	0.53 ± 0.01
	50	3.32 ± 0.01	1.28 ± 0.01	1.34 ± 0.01	1.86± 0.01	0.72 ± 0.01	0.75 ± 0.01
	75	4.23 ± 0.06	1.61 ± 0.02	1.86 ± 0.01	2.23 ± 0.01	0.85 ± 0.01	0.98 ± 0.01
	100	4.63 ± 0.04	1.86 ± 0.02	2.29 ± 0.01	2.27 ± 0.02	0.91 ± 0.01	1.13 ± 0.01
Pb sheet		63.06 ± 0.05	25.99 ± 0.13	29.59 ± 0.03	5.56 ± 0.01	2.25 ± 0.01	2.61 ± 0.01

**Table 4:** Recommended Bi_2_O_3_ contents in WPVC composites containing 20 pph and 40 pph wood particles for attenuation of X-rays generated from a tube with supplied voltages of 60, 100, and 150 kV.

X-ray Tube Voltage (kV)	Recommended Bi_2_O_3_ Content (pph)	
	20 pph Wood	40 pph Wood
60	35	40
100	85	100
150	40	45

**Table 5:** Comparative X-ray shielding properties of WPVC and other composites containing Bi_2_O_3_ as X-ray protective filler.

Matrix	Bi_2_O_3_ Content	X-ray Energy	µ_m_ (cm^2^/g)	Reference
WPVC	100 pph (35 wt.%)	100 kV (~40–60 keV)	0.91–1.01	This work
Natural rubber latex (NRL)	100 phr (50 wt.%)	80 kV (~30–50 keV)	1.32	[12]
Silicone rubber (SR)	50 wt.%	55 keV	1.01–1.20	[52]
SR	50 wt.%	59 keV	0.08–0.22	[53]
SR	50 wt.%	66 keV	0.00–0.44	[53]
Epoxy	35 wt.%	59.54 keV	1.343	[54]

## Data Availability

The data presented in this study are available on request from the corresponding author.
